# A hybrid filtering and deep learning approach for early Alzheimer’s disease identification

**DOI:** 10.1038/s41598-025-03472-z

**Published:** 2025-07-29

**Authors:** Md. Khabir Uddin Ahamed, Rakib Hossen, Bikash Kumar Paul, Mohammad Hasan, Waled Hussein Al-Arashi, Mohsin Kazi, Md. Alamin Talukder

**Affiliations:** 1 Department of Computer Science and Engineering, Jamalpur Science and Technology University, Jamalpur, Bangladesh; 2 Department of Cyber Security Engineering, Gazipur Digital University, Kaliakair, 1750 Bangladesh; 3 Department of Computer Science, Virginia Tech, Blacksburg, 24060 USA; 4 Department of Computer Science and Engineering, Jamalpur Science and Technology University, Jamalpur, Bangladesh; 5 Faculty of Engineering and Computing, University of Science and Technology, Aden, Yemen; 6 Department of Pharmaceutics, College of Pharmacy, King Saud University, P.O. Box-2457, Riyadh, 11451 Saudi Arabia; 7 Department of Computer Science and Engineering, International University of Business Agriculture and Technology, Dhaka, Bangladesh; 8 School of Information Technology, Crown Institute of Higher Education, Canberra, Australia

**Keywords:** Alzheimer’s disease, Diagnosis, Transfer learning, MRI-image, EfficientNetV2B3, Grad-CAM++, Cancer imaging, Diseases, Computer science

## Abstract

Alzheimer’s disease is a progressive neurological disorder that profoundly affects cognitive functions and daily activities. Rapid and precise identification is essential for effective intervention and improved patient outcomes. This research introduces an innovative hybrid filtering approach with a deep transfer learning model for detecting Alzheimer’s disease utilizing brain imaging data. The hybrid filtering method integrates the Adaptive Non-Local Means filter with a Sharpening filter for image preprocessing. Furthermore, the deep learning model used in this study is constructed on the EfficientNetV2B3 architecture, augmented with additional layers and fine-tuning to guarantee effective classification among four categories: Mild, moderate, very mild, and non-demented. The work employs Grad-CAM++ to enhance interpretability by localizing disease-relevant characteristics in brain images. The experimental assessment, performed on a publicly accessible dataset, illustrates the ability of the model to achieve an accuracy of 99.45%. These findings underscore the capability of sophisticated deep learning methodologies to aid clinicians in accurately identifying Alzheimer’s disease.

## Introduction

As the global population ages, the prevalence of Alzheimer’s disease (AD), an irreversible, relentless neurodegenerative disorder, continues to obliterate memory and cognitive function, presenting a grave peril that could ultimately lead to death^1^. Early Alzheimer’s disease diagnosis pertains to identifying the condition during its nascent phases, before the onset of substantial cognitive deterioration, to implement medical interventions that can extend daily functioning^2^. According to^3^, AD currently impacts an estimated 36.6 million individuals; this figure is projected to double within the following two decades.^4^ mention that AD has a global impact, affecting an estimated 46 million individuals; however, precise figures regarding the exact number of those affected remain unavailable. Nevertheless, in previous times, diagnostic techniques, which frequently depended on subjective clinical evaluations, encountered constraints regarding precision and effectiveness. Additionally, the overall expense and effort incurred by patients and their families are escalating substantially^5,6^.

Recent advancements in deep learning (DL) models have shown great promise in improving the accuracy and efficiency of Alzheimer’s disease detection. These models leverage large datasets, including medical imaging and clinical data, to identify subtle patterns and biomarkers associated with the disease at its earliest stages. By automating and enhancing diagnostic processes, DL-based approaches offer a potential solution to the limitations of traditional methods. However, in recent years, the performance of image-based Alzheimer’s disease detection using pre-trained convolutional neural network models has been superior to existing methods^7^. Numerous advantages underscore the potential of deep learning as a promising instrument for disease diagnosis and medical image analysis, with the capacity to fundamentally transform the domain of Alzheimer’s detection^8^. This study evaluates and proposes an innovative deep-learning approach for the early detection of Alzheimer’s disease to address this critical issue. Scholars are in quest of a straightforward and precise method to diagnose Alzheimer’s disease before the onset of symptoms. Early Alzheimer’s detection identifies symptoms before the disease progresses to the risk stage^9^. Conventional diagnostic approaches are laborious and subjective and might overlook subtle subtleties that are suggestive of Alzheimer’s pathology in its early stages. As a result, sophisticated diagnostic instruments that can improve the precision and effectiveness of detection are urgently required. As a result, the significance of non-invasive biomarkers and the transformative potential of artificial intelligence and deep learning in early AD detection continues to grow^10^. Deep learning, classified as a subset of artificial intelligence, can significantly alter medical imaging by focusing on its effects on segmentation, object detection, and image classification^11,12^ across multiple medical domains. In particular, deep convolutional neural networks (CNNs) demonstrate exceptional proficiency in deriving intricate features and patterns from complex datasets. Deep hypercomplex-inspired CNNs enhance feature extraction for image classification by allowing weight sharing across input channels^13^. Researchers have developed efficient deep-learning models that can accurately detect features from sizeable medical image datasets without using deeper layers to detect Alzheimer’s disease in its early stages^14,15^.

The study aims to address current diagnostic challenges related to Alzheimer’s disease (AD) and enhance diagnostic accuracy through deep learning capabilities. A powerful tool will be provided to clinicians to enable earlier interventions and ultimately improve the quality of patient care. Moreover, this research not only makes a valuable contribution to the progression of AI-powered healthcare but also has the potential to assist physicians in the early detection of Alzheimer’s disease, thereby ultimately enhancing the well-being of those afflicted.

### Contribution of the proposed study

Here is a summary of the primary contributions:

i. A novel Hybrid Filtering approach was developed to pre-process the image data to facilitate accurate and efficient analysis by our proposed deep transfer learning framework.
ii. An advanced deep transfer learning model was designed based on EfficientNetV2B3. The model involves attaching layers with fine-tuning to enable rapid and precise identification and diagnosis of Alzheimer’s disease cases.
iii. An explainable AI technique was explored and employed, utilizing Grad-CAM++ for discriminative localization to mark the most reliable classification results of Alzheimer’s disease patients using MRI image datasets and improving the model transparency and interpretability.

***Research questions***

- How well does the proposed hybrid filtering method enhance Magnetic Resonance Imaging (MRI) image preprocessing to strengthen model robustness and improve accuracy?
- How does the proposed model perform relative to other state-of-the-art methods regarding accuracy, precision, recall, and specificity for multi-class Alzheimer’s disease diagnosis?
- How do advanced explainable AI techniques, such as Grad-CAM++, enhance clinicians’ ability to interpret and trust AI-based diagnostic approaches?

The following sections of this article are organized in the following manner: section "Literature review" offers a thorough summary of the research conducted in the study area. Section "Proposed methodology" outlines the methodology of the proposed work. Section "Result analysis" presents experimental results and describes the dataset. Section "Discussion" discusses the suggested study. Section "Conclusions" contains our results and recommendations for future development efforts.

## Literature review

Alzheimer’s disease is a progressive neurological condition caused by the deterioration of nerve cells in different brain areas^16^. It poses significant physical and psychological challenges to both patients and their families. Individuals experience behavioral, emotional, and physical declines as the disease advances. Although the complete cure is a concerning issue, early detection is crucial for managing the condition effectively. In recent years, learning-based methods like deep and machine learning have significantly advanced the early detection of various diseases, including heart, eye, lung, and breast cancer^17–20^. These techniques have also been extensively applied to the preliminary diagnosis of Alzheimer’s disease, enhancing early identification and intervention strategies.

### Limitations of existing diagnostic methods

Despite progress in the comprehension of Alzheimer’s disease, numerous limitations remain in existing diagnostic methods. Conventional approaches typically diagnose Alzheimer’s disease only after the onset of substantial symptoms and considerable brain damage, thereby restricting the efficacy of interventions^21^. These methods frequently depend on cognitive assessments and patient history, demonstrating limited predictive capability concerning disease progression^21^. Although biomarkers in cerebrospinal fluid (CSF) and amyloid PET scans can enhance diagnostic accuracy, these techniques are intrusive, expensive, and not readily available^22^. Ziyad et al.^23^ highlight the importance of AI in detecting Alzheimer’s, achieving 97% accuracy in classifying patients using CNNs on MRI scans. This approach aims to enhance prognosis through early detection.^24^ have embraced a more comprehensive viewpoint, contending that early detection of Alzheimer’s disease through the utilization of MRI images in conjunction with support vector machine (SVM) and transfer learning technologies yields an improved accuracy rate of 94.44%. This hybrid architecture integrates the LSTM and Deep Maxout neural networks. The study by^25^ offered probably the most comprehensive empirical analysis of a machine learning model that comprised GaussianNB, Decision Tree, Random Forest, XGBoost, Voting Classifier, and GradientBoost to predict Alzheimer’s disease. With a validation accuracy 96% for the AD dataset, the model is trained utilizing the open access series of imaging studies (OASIS) dataset. As they pertain to AD detection, deep learning and machine learning were examined in the article by^26^. Utilizing Group Grey Wolf Optimization (GGWO) techniques enhanced the efficacy of CNN, KNN, and decision tree classifiers, achieving 96.23% accuracy in Alzheimer’s detection compared to other methods. Employing convolutional and recurrent neural networks,^27^ put forth a classification system designed to address Alzheimer’s disease. Because of this, we need non-invasive, rapid, affordable, and accurate ways to diagnose AD right away.

### Deep learning models for enhanced Alzheimer’s disease detection

The subject of Alzheimer’s disease analysis has been dramatically enhanced by recent developments in interpretable deep-learning models. For example,^28^ explores contemporary perspectives on machine learning in the context of precision psychiatry, highlighting the significance of model interpretability and personalized predictions. These findings emphasize the crucial requirement for creating models that attain high precision and provide insight into the fundamental mechanisms of Alzheimer’s disease. By integrating interpretability-focused approaches, the clinical applicability of deep learning models can be significantly enhanced, guaranteeing that they offer healthcare providers practical and valuable insights.

Table1 presents some recent state-of-art works on Alzeimir’s disease detection. Hazarika et al.^29^ introduced a hybrid model that merges LeNet and AlexNet, applied to the ADNI dataset, and achieved an accuracy of 93.58%, noting that many models struggle to surpass 90% in classification tasks. Meanwhile, Mawra et al.^30^ developed a CNN architecture with the OASIS dataset, reaching 99.68% accuracy, but pointed out challenges related to generalizability and interpretability. Chethana et al.^31^ used a combination of CNN and RNN on the ADNI dataset, achieving 98.45% accuracy, with a primary concern being the potential for overfitting due to model complexity. Suchitra et al.^32^ employed the EfficientNetB7 model on the ADNI dataset, achieving 98.2% accuracy in Alzheimer’s disease classification. However, the study’s limitations include a lack of interpretability due to the model’s complexity. Valoor and Gangadharan^33^ utilized a hybrid U-net and GAN model to analyze data from the ADNI dataset, achieving an accuracy of 95%. Their approach demonstrated promising results in medical imaging applications but faced scalability challenges when applied to multi-modal data. Similarly, Kina^34^ proposed an attention-based deep learning model, analyzing data from Kaggle with an accuracy of 95.19%. However, the model exhibited suboptimal performance in handling specific subsets of the dataset, indicating room for improvement in generalization. Both studies underscore the effectiveness of advanced DL architectures while highlighting the need to address challenges like scalability and dataset-specific limitations for broader applicability.

### The role of explainable AI (XAI)

Explainable AI (XAI) techniques are essential for addressing this gap, offering insights into the decision-making processes of AI models and improving transparency^35^. Some investigations used explainable AI (XAI) to analyze MRI images of Alzheimer’s disease cases visually. ÖZBAY, F. A et al.^36^ proposed NCA-based CNN models to detect Alzheimer’s disease, where for the discriminative visualization they used Grad-CAM (Gradient-weighted Class Activation Mapping). Chethana, S. et al.^31^ suggested an approach employing Convolutional Neural Networks (CNN) and Recurrent Neural Networks (RNN) on MRI scans to classify AD stages. Additionally, Explainable AI (XAI) via Grad-CAM is utilized to identify affected brain regions, addressing diagnostic delays. Mahmud et al.^37^ utilized ensemble deep learning models on the Kaggle dataset, achieving accuracies up to 96%. Their approach incorporated explainable AI techniques (saliency maps and grad-CAM) to enhance the model’s interpretability and provide insights into the diagnostic process. A limitation noted was the potential for biases within the datasets used.

### Problem statement and research gap

Most studies mentioned above utilized a relatively small number of Alzheimer’s disease cases to train different machine-learning models. Small datasets are prone to causing overfitting in a convolutional neural network (CNN) framework. The model would not accurately reflect classification performance on datasets not included in the training data. Moreover, several current techniques are trained and evaluated using raw images without preliminary processing or augmentation. Therefore, the network’s generalization error rises while the training benefits are reduced. Furthermore, most studies have utilized pre-trained models and trained their frameworks with either three or two classes. Some studies used conventional explainable AI techniques for discriminative localization.

To address these challenges, we constructed a substantial and reliable dataset. We then conducted thorough novel pre-processing and augmentation procedures on the gathered images rather than utilizing them in their original state. Our study enhanced a classic deep transfer learning model by fine-tuning and optimizing hyperparameters to increase model robustness. The study was improved by combining multi-class image class comparisons using four-class categories. We explored and utilized an advanced explainable AI called Grad-CAM++ for the discriminative localization of the resulting images to increase the model interpretability and generability.

**Table 1:** Some recent state-of-the-art works in the field of Alzheimer’s disease detection.

Author (s) & Year	Work done	Dataset	Performance	Visualization	Limitations
			Metrics	with XAI	
^29^	Hybrid model merging	ADNI	Hybrid model achieved	x	Most current models performed
	LeNet and AlexNet		93.58% accuracy		less than 90% in classification task
^38^	CNN and LSTM	Kaggle	Attained an accuracy	x	Weight decay issues may arise,
			of 98.5%		optimal solution does not cover
^39^	VGG-16-based CNN	ADNI	Achieved an accuracy	x	Classifying pMCI and sMCI is
	with Transformer		of 77.2%		difficult due to subtle differences
^40^	Customized AlexNet &	ADNI	Gained accuracy of 96.61%	x	Model’s interpretability &
	InceptionV2 architecture		and AUC of 0.9663		explainability limited
^30^	CNN architecture	OASIS	Achieved an accuracy of	x	Lack of generalizability &
			99.68%		interpretability
^14^	Lightweight DL Model	Kaggle	Achieved an accuracy of	x	Potential overfitting due to
			95.93%		the small dataset size
^32^	Enhanced EfficientNetB7	ADNI	Achieved an accuracy of	x	Lack of interpretability
			98.2%		
^36^	Densenet201, EfficientNet	Kaggle	Achieved an accuracy of	Yes	Combination of different TL
	and AlexNet		99.83%		prone to rise complexity
^31^	CNN with RNN	ADNI	Achieved an accuracy of	Yes	Potential for overfitting due
			98.45%		to complex models
^37^	Ensemble DL models	Kaggle	Achieved an accuracy of	Yes	Potential biases in dataset
			96%		
^33^	U-net+GAN	ADNI	Achieved an accuracy of	Yes	Scalability challenges with multiple models
			95%		
^34^	Attention DL model	Kaggle	Achieved an accuracy of	Yes	Subpar performance in specific cases
			95.28%		

## Proposed methodology

**Figure 1:**
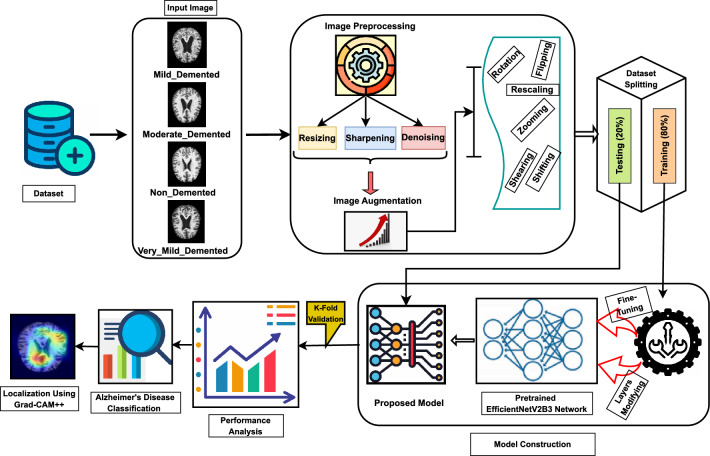
Proposed workflow diagram of Alzheimer’s disease classification.

This section delineates the processes of data preprocessing, data augmentation, the CNN model, and the implemented architecture that were carried out before conducting trials for performance evaluation. Figure 1 visually represents the proposed methodology. The proposed Alzheimer’s Disease detection system utilizes MRI scans of the human brain as input in this block diagram. Subsequently, the gathered images undergo preprocessing by applying resizing and denoising methods. Subsequently, the image data undergoes augmentation. Ultimately, the model performed training and testing using the clinical datasets. The proposed model was constructed using the foundational deep learning model termed EfficientNetV2B3. This study involved the development of the model by incorporating additional layers into its underlying network. The layers experienced modifications through regularization, kernel initialization, and reliable fine-tuning techniques to enhance the experiment’s robustness and efficiency. Furthermore, this model can precisely categorize images into four distinct classes with the maximum level of accuracy. Again, the pseudocode of AD classification from MRI scans is illustrated in Algorithm 1.

**Algorithm 1:**
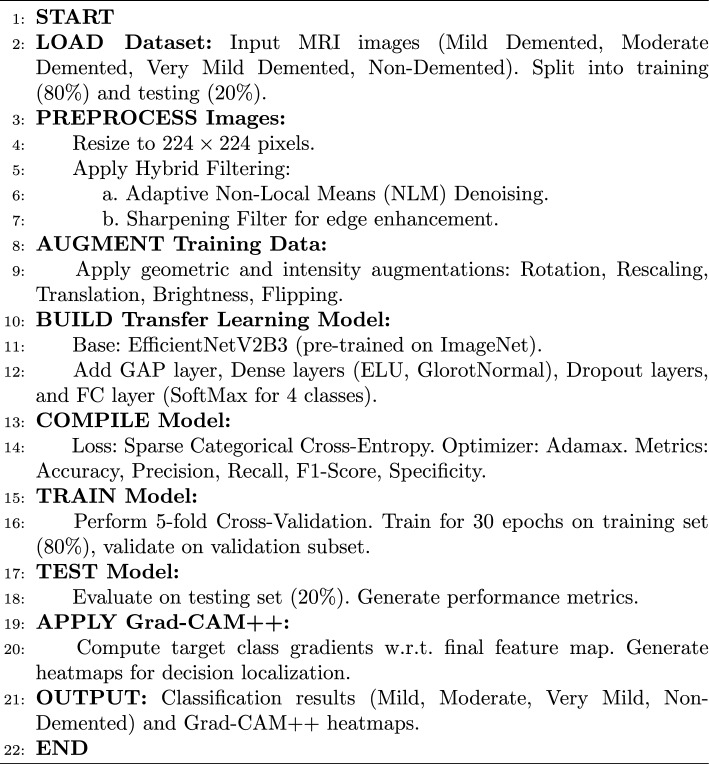
Pseudocode of Alzheimer’s Disease Classification from MRI Images

### Preprocessing of images

Preprocessing the images from the dataset is essential to optimizing the learning module’s performance and ensuring accuracy. This preprocessing phase consists of effective resizing with hybrid filtering (adaptive nonlocal means denoising and sharpening filter) operations on the image data.

#### Resizing of images

The inputs’ dimensions over the models pre-trained on ImageNet will be either smaller or equal to 224*224. For transfer learning, it is necessary to ensure that the inputs are compatible with the pre-trained model. To ensure uniform dimensions for all collected images, we resized individuals to a resolution of 224*224 pixels.

#### Denoising of images

Denoising an image involves the restoration of a signal from images that contain noise. Denoising is eliminating undesirable noise from an image to enhance its analysis. One effective method for image denoising is the Non-Local Means (NLM) technique. Non-Local Means (NLM) denoising is a widely used method for reducing noise in images^41^. It operates on the principle that patches in an image with comparable characteristics also have identical pixel values. NLM denoising is a technique that averages pixel values of similar patches in an image to reduce noise while preserving underlying structures, using a weighted average based on similarity between the reference patch and neighborhood patches.

We may now mathematically represent this process. Let’s consider *I* as the image affected by noise, where *u* denotes the pixel values in the noisy image, and *v* represents the pixel values after the denoising process.

The Equation1 for NLM denoising is expressed as :

1 $$\begin{aligned} v(x) = \frac{1}{C(x)} \sum _{y \in \Omega } w(x, y) \cdot u(y) \end{aligned}$$

where *x* denotes the position of a pixel within the image, while *y* represents the pixel’s position in the neighboring region $\Omega$ centered around *x*. The function *w*(*x*, *y*) assigns a weight to the pixel at location *y*, which is determined by the similarity of the patches surrounding pixels *x* and *y*. *C*(*x*) serves as a normalization factor, ensuring that the weights assigned to pixels around *x* sum up to 1, maintaining the integrity of the weighted averaging process. The weight function *w*(*x*, *y*) can be defined by utilizing a Gaussian kernel in Equation2:

2 $$\begin{aligned} w(x, y) = \frac{1}{Z(x)} e^{-\frac{\Vert P(x) - P(y)\Vert ^2}{h^2}} \end{aligned}$$

where the patch located at point *x* is denoted as *P*(*x*). The decay of weights can be controlled using the parameter *h*. The normalization coefficient *Z*(*x*) ensures that all the weights add up to 1. The NLM denoising approach entails traversing all pixels in the image and implementing the weighted averaging procedure to efficiently remove noise from the image.

Reducing noise in MRI images is essential for enhancing diagnostic precision and minimizing artifacts that could hinder interpretation. Traditional methods like Non-Local Means (NLM) filtering can be slow and may not adapt well to images’ diverse noise characteristics and intensity variations. We employed the Adaptive Non-Local Means (NLM) filter approach in our investigations to address this. This technique dynamically adjusts filtering strength based on local image properties, guaranteeing optimal noise reduction across various intensities.

**Figure 2:**
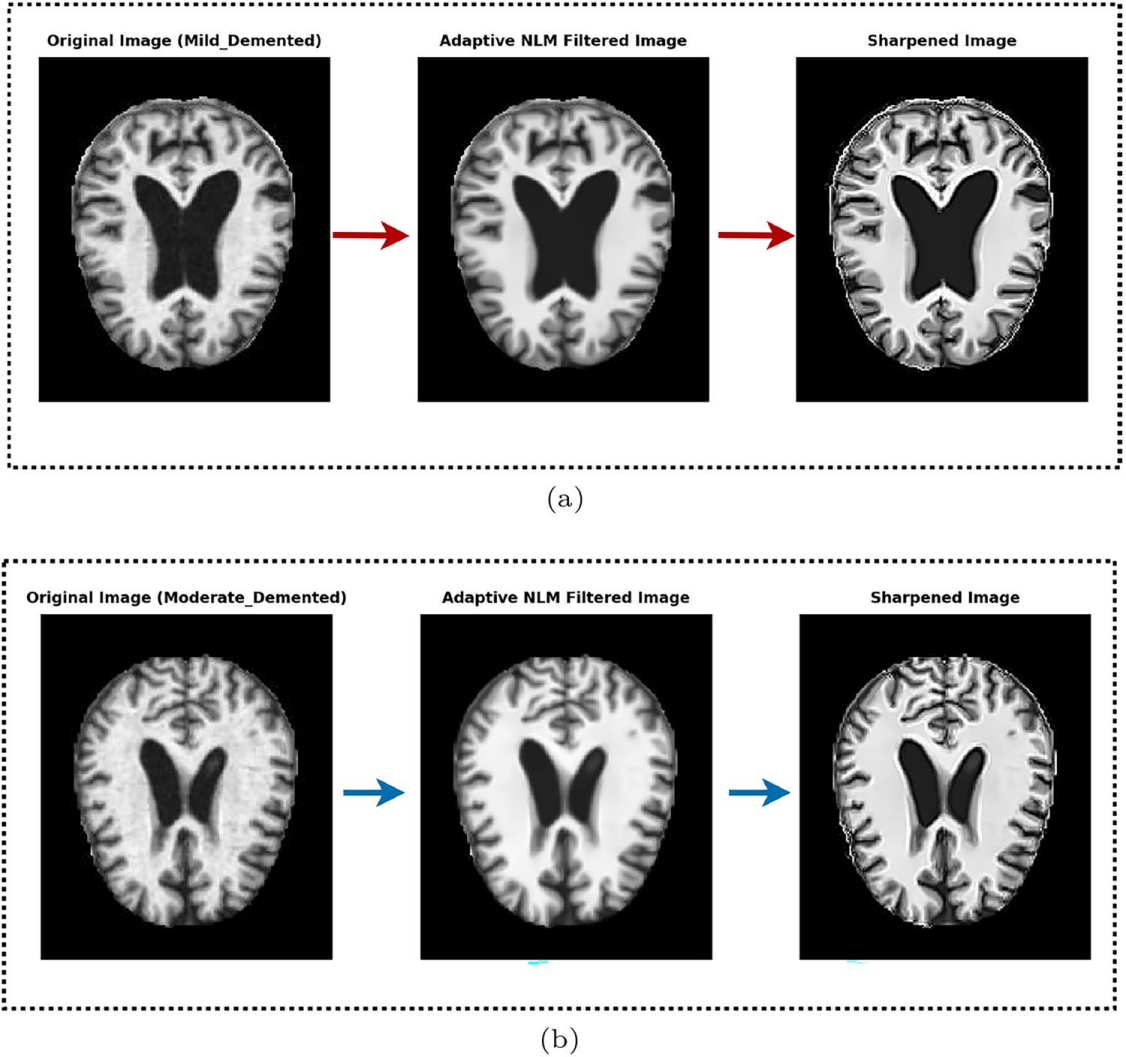
Sample preprocessing results based on Adaptive NLM Filter and Sharpening Filter: (**a**) Mild_demented case (**b**) Moderate_demented case.

In particular, the method initially computes the mean intensity of the input image. This means intensity is then used to adjust the filtering parameter *h* in the cv2.fastNlMeansDenoising function from the OpenCV library. The adjustment is based on the following relationship as shown in Equation3.

3 $$\begin{aligned} h = \text {base}\_\text { h} \times (1 + \text {mean}\_\text {intensity}) \end{aligned}$$

where base $\_h$ is a predetermined baseline value, and in our study, the baseline value 10 was used in this case. When the mean intensity is high, *h* increases, enhancing filtering to manage higher noise in brighter areas. In contrast, darker regions undergo milder filtering to retain details that uniform filtering might obscure. The fastNlMeansDenoising function uses templateWindowSize set to 7 and searchWindowSize set to 21, determining the local neighborhood sizes for filtering. Before filtering, the input image is normalized to a [0, 255] range and then scaled back to its original range. This adaptive technique effectively reduces noise while preserving fine details. Figure 2 shows the preprocessing results of using the Adaptive NLM filter.

#### Sharpening of images

Following the noise reduction procedure, a sharpening filter is incorporated in this study to enhance the collected MRI image details and improve edge definition. The non-local means (NLM) filtering inherent in the denoising process can cause blurring, potentially obscuring fine details and softening edges. A subsequent sharpening filter mitigates this blurring, enhancing the visibility of subtle features and improving the overall image sharpness. This sharpening step is beneficial for tasks such as feature extraction, where precise edge definition is crucial.

The concept of the sharpening filter is based on Laplacian filters, which emphasize areas of rapid intensity change and represent a second-order derivative enhancement system^42^. Typically, this is derived as shown in Eq. 4.

4 $$\begin{aligned} \nabla ^2 f = \frac{\partial ^2 f(x,y)}{\partial x^2} + \frac{\partial ^2 f(x,y)}{\partial y^2} \end{aligned}$$

Here,

5 $$\begin{aligned} \frac{\partial ^2 f(x,y)}{\partial x^2} \approx f(x+1,y) + f(x-1,y) - 2f(x,y) \end{aligned}$$

6 $$\begin{aligned} \frac{\partial ^2 f(x,y)}{\partial y^2} \approx f(x,y+1) + f(x,y-1) - 2f(x,y) \end{aligned}$$

Equation 4 leads to the derivation of the discrete Laplacian:

7 $$\begin{aligned} \nabla ^2 f \approx f(x+1,y) + f(x-1,y) + f(x,y+1) + f(x,y-1) - 4f(x,y) \end{aligned}$$

**Figure 3:**
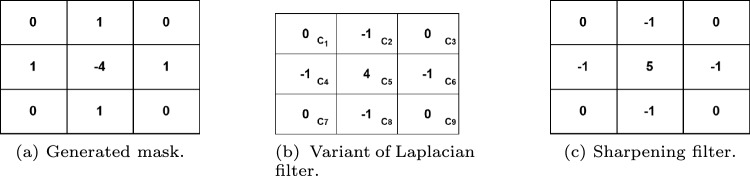
An illustration of various filters is presented: (**a**) the generated mask, (**b**) a modified Laplacian filter, and (**c**) a sharpening filter.

From Eq. 7, a mask is generated as presented in Fig. 3a. Additionally, the literature^43^ indicates that various other types of Laplacian masks and filters are available. This study employs a modified Laplacian filter, the kernel shown in Fig. 3b. Hence, the intensity value in the given Laplacian filter is calculated by adding the center point of the mask to the sum of the surrounding points. This can be expressed as:$C5 + (C1 + C2 + C3 + C4 + C6 + C7 + C8 + C9)$. In this case, the intensity value of “0” is obtained by summing the center value with the surrounding values in the mask. When the original image is processed using this mask, the result is a darker image that highlights only the edges, where the intensity value is 0. The original image can be reconstructed using the rules specified in Eqs. 8 and 9.

8 $$\begin{aligned} g(x,y)=f(x,y)-\bigtriangledown ^{2}f;W_5<0 \end{aligned}$$

9 $$\begin{aligned} g(x,y)=f(x,y)+\bigtriangledown ^{2}f;W_5>0 \end{aligned}$$

In this context, *g*(*x*, *y*) denotes the filtered output after applying the desired operation. If the center value of the Laplacian filter is negative, the process follows Eqs. 8 and 9; otherwise. Let us define *f*(*x*, *y*) as *C*5, with $C1,C2,\ldots ,C9$ representing the other surrounding points. Based on the corresponding Laplacian mask, Eq. 10 can be derived from Eq. 9. From Eq. 10, the resulting mask or filter is shown in Fig. 3c.

10 $$\begin{aligned} g(x,y)=9W_5-W_1-W_2-W_3-W_4-W_6-W_7-W_8-W_9 \end{aligned}$$

This filter, known as the sharpening filter, is designed to enhance image details and emphasize edges. The sharpening filter process is implemented using OpenCV’s filter2D function. In addition, it helps make transitions between features more distinct and noticeable, in contrast to smooth, noisy, or blurred images. Figure 2 depicts the preprocessing results of using the Sharpening filter.

### Augmentation of images

Image augmentation^44^ is a widely used technique in deep learning that artificially enhances the training dataset’s variety through various modifications to the source images. This contributes to the improvement and resilience of deep learning models. Augmentation techniques commonly encompass operations that involve rotation, mirroring, scaling, cropping, adjustments in the brightness and contrast and other geometrical or color alterations can be operated by using ImageDataGenrator^45^. So, applying image augmentation procedures in our study, we can mitigate overfitting and enhance the model’s capacity to handle diverse real-world situations, resulting in a more efficient and dependable model for our proposed deep learning model. The augmentation approaches employed in our investigation are presented in Table 2 and the description of these augmentation criteria is given as follows:

**Table 2:** Augmentation criteria and value distribution.

Augmentation criteria	Value
Rotation_range	7
Width_shift_range	0.05
Height_shift_range	0.05
Zoom_range	0.1
Rescale	1./255
Shear_range	0.05
Brightness_range	[0.1, 1.5]
Horizontal_flip	True
Vertical_flip	True

- Rotation: Applied random rotations across a range of ${\pm }7$ degrees to achieve model invariance to varying orientations of brain scans.
- Width and Height Shifting: Horizontal and vertical transformations of up to 5% of the image sizes were employed to replicate positional variation in MRI images.
- Zooming: A zoom range of 10% was utilized to simulate variations in the field of view.
- Rescaling: All pixel values were normalized into the interval [0, 1] by dividing by 255, enhancing convergence during training.
- Shearing: A shear transformation with a magnitude of 5% was used to induce affine distortions, hence improving the model’s generalization capacity.
- Brightness Adjustment: Random brightness fluctuations within the interval [0.1, 1.5] were implemented to accommodate variations in image contrast and lighting circumstances.
- Flipping: Horizontal and vertical flips were utilized to emulate various views, hence diminishing bias towards particular orientations.

### Proposed transfer learning model

The proposed model methodology relies on a sophisticated deep transfer learning (DTL) architecture. Scholars have recently shown a growing interest in employing transfer learning-based convolutional neural network (CNN) models to address diverse computer vision challenges. These models have gained extensive application in medical disease diagnostics^11,19^ over the past few decades. In this study, we developed and implemented a transfer learning architecture based on CNNs to classify Alzheimer’s disease from MRI images. In our experiment, we train a DTL pre-trained CNN model termed EfficientNetV2B3 using the preprocessed image data. The DTL algorithm that was adopted in our experiment is explained below:

- EfficientNetV2B3 : EfficientNetV2B3 is a deep neural network architecture introduced as an extension of the EfficientNet family^46^. The EfficientNetV2B3 model is designed to optimize computational efficiency and model performance across various tasks, particularly image classification. It incorporates improvements over its predecessors, including enhanced feature extraction capabilities and a well-balanced trade-off between model size and accuracy. This architecture is based on compound scaling, which uniformly scales the network dimensions (depth, width, and resolution) to achieve better performance. EfficientNetV2B3 is pre-trained on large-scale datasets, making it effective for transfer learning tasks. Its architecture allows for efficient utilization of computational resources while maintaining competitive accuracy levels.

Therefore, we choose EfficientNetV2B3 rather than EfficientNetV2B0, B1, and B2 because EfficientNetV2B3 is anticipated to possess a more extensive architecture when compared to EfficientNetV2B0, B1, and B2. The heightened depth is expected to enhance the model’s capacity for capturing intricate features, potentially leading to improved capabilities in representation learning. Additionally, EfficientNetV2B3 is projected to have an increased width, leading to a more expansive capacity for feature extraction. Furthermore, EfficientNetV2B3 will likely feature an elevated resolution compared to its forerunners. This heightened resolution has the potential to augment the model’s capability to capture intricate details in images, enhancing its overall performance.

#### Design and modification of the model’s structure

The proposed study used EfficientNetV2B3 as the foundational model. The base network was constructed and developed using techniques including layer attachment, regularization, kernel initializer, and hyper-parameter tuning.

**Figure 4:**
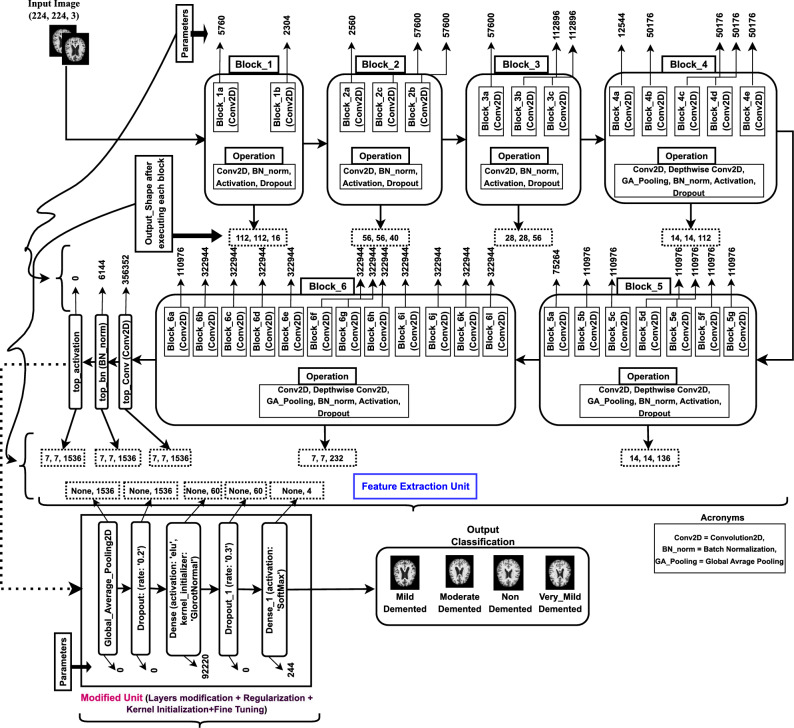
The proposed transfer learning architecture, consisting of: Feature Extraction unit (including Block-wise operations, parameters, output-shape) and Modified Unit (including layers modification, regularization, kernel initialization, hyper-parameter tuning, parameters and output-shape).

The model takes a pre-processed dataset of images with dimensions of 224*224 pixels as input. Figure 4 depicts the block-wise internal details of the EfficientNetV2B3 pre-trained network. The feature extraction unit of EfficientNetV2B3 has six blocks that incorporate essential operations, including convolution, depth-wise convolution, batch normalization, activation, dropout, and global-average pooling. Block 1, block 2, block 3, block 4, block 5 and block 6 have two, three, three, five, seven and twelve sub-blocks with out-shape (112, 112, 16), (56, 56, 40), (28, 28, 56), (14, 14, 112), (14, 14, 136) and (7, 7, 232) respectively. Block 1a contains a total of 5760 parameters. Upon completing six blocks, Block 6l generates 322944 parameters respectively.

After the EfficientNetV2B3 model completes its six blocks, which progressively reduce spatial dimensions and increase the number of channels, the final feature extractor layer outputs a $7 \times 7 \times$ 2048 feature map. This map is then processed by a global average pooling layer, which reduces the spatial dimensions to a $1 \times 1$ grid. This pooling layer averages all values within each feature map, simplifying the model and enhancing robustness by reducing the risk of overfitting. Subsequently, dense layers with the ELU activation function and GlorotNormal kernel initializer are added, along with dropout layers to prevent overfitting. The model concludes with a fully connected layer using the SoftMax activation function for multiclass classification. This configuration enables the model to utilize the detailed feature representations from the EfficientNetV2B3 backbone while maintaining a straightforward and efficient classification mechanism.

#### Model development process

- Layer Modification and Fine-tuning: Following the final activation layer of the pre-trained network, we incorporated global average pooling, dense layers with the eLU activation function, and “GlorotNormal” kernel initializer, dropout layers and the modification was ended with a fc (fully connected) layer named SoftMax for multiclass categorization. We used fine-tuning to make it easier for the pre-trained models to adapt to our particular task. The following points give more details of our model development:
  i. GA Pooling Layer: The Global Average Pooling (GAP) layer is integral to convolutional neural networks (CNNs) for image classification. Unlike traditional fully connected layers, GAP operates on the entire spatial dimensions of the input feature map. Computing the average of all values within each feature map condenses information, resulting in a single value per feature map and effectively reducing spatial dimensions to a 1x1 grid. While GAP can partially provide translation invariance by limiting the spatial dimensions of the feature map to a single value per feature map, it does not entirely ensure translation invariance. The main objective of incorporating GAP (Global Average Pooling) into our architecture is to reduce the number of model parameters and mitigate the risk of overfitting. This, in turn, can improve our system’s resilience to slight spatial translations.
  ii. Dropout Layer: The Dropout layer is a regularization technique employed in neural networks to mitigate overfitting. During training, it randomly deactivates a specified fraction of neurons, preventing co-dependency and encouraging the network to learn more robust features. This dropout of neurons simulates training multiple diverse networks and improves the model’s generalization capabilities. Randomly excluding neurons using a Dropout layer in our study reduces the risk of overfitting, enhancing the proposed model’s ability to adapt to various inputs and improving its overall performance on unseen data. The proposed study incorporates two dropout layers, each having a dropout rate of 0.2 and 0.3, respectively.
  iii. Dense Layer: Dense layer also known as a fully connected layer, is a fundamental component in neural networks. It connects every neuron from one layer to every neuron in the subsequent layer, creating a dense interconnection pattern. Each connection is associated with a weight and the layer typically includes a bias term. The Dense layer plays a pivotal role in our study for learning complex patterns and relationships within the data. Our study structures the dense layer with the ’eLu’ activation function and the ’GlorotNormal’ kernel initializer.
    The “Glorot Normal” kernel initializer, sometimes referred to as Xavier normal initialization, aims to mitigate the problems of vanishing or exploding gradients by sampling weights from a normal distribution with a mean of 0 and a variance that depends on the number of input and output units. This method guarantees that the weights are evenly distributed, enhancing the workout’s stability and effectiveness. Although the tanh activation function is often linked to it, its usefulness extends beyond just tanh. The initializer is effective with the eLU activation function since it may ensure a consistent and steady flow of gradients.
    The eLU activation function mitigates the vanishing gradient problem, a prevalent challenge in deep networks, and permits negative inputs, hence minimizing the occurrence of dead neurons. The smooth gradient of the training process is enhanced by the “Glorot Normal” initializer, resulting in stable and efficient training. The effectiveness of this combination was confirmed by empirical validation in our experiments, exhibiting enhanced performance and stability during the training process.
    The objective is to utilize the “Glorot Normal” initializer and the eLU activation function to improve our model’s learning capacity and stability.
  iv. SoftMax Activation Layer(Output Classification): We utilized an output layer activated with softmax for the classification task. This layer assigns probabilities to diverse classes, enabling the efficient categorization of MRI images into their respective relevant groups.
  v. Random Seed: Employing a constant random seed is crucial for guaranteeing the replicability of our studies. By specifying the seed as 45, we ensure that every execution of our model will yield consistent outcomes. Reproducibility is crucial in scientific study, enabling us to compare our findings with other investigations.
  vi. Adamax Optimizer: Adamax, an extension of the Adam optimizer for deep learning, employs adaptive learning rates, dynamically adjusting them during training. It utilizes the infinity norm for stable parameter updates and is characterized by two key parameters, beta1 and beta2, controlling moment estimate decay rates. Adamax excels in handling sparse gradients, demonstrating robust performance across various neural network architectures. To ensure numerical stability in the parameter update step, a small positive constant, epsilon $\epsilon$, is added to the denominator, preventing division by zero. This feature enhances the reliability and efficiency of our research optimization processes, so Adamax is a popular choice in our proposed deep neural network training. In our investigation, we employed a learning rate of 1e-03 and a decay rate having two parameters: beta1=0.91 and beta2=0.9994. We also set the epsilon value to 1e-08.

## Result analysis

### Dataset description

The dataset employed for the investigation was obtained from a publicly accessible source. The collection comprises 6400 MRI images of Alzheimer’s disease in total. These images were obtained from Kaggle’s “Alzheimer’s Dataset (4 classes of Images)” by Yasir Hussein Shakir^47^. We chose this dataset because it is publicly available, sufficiently sized for deep learning, and includes multi-class categorization of Alzheimer’s stages, allowing for a detailed investigation of disease progression. While acknowledging the class imbalance, especially in the Moderate Demented group, we used data augmentation and regularization strategies to reduce its influence and strengthen the model.

**Figure 5:**
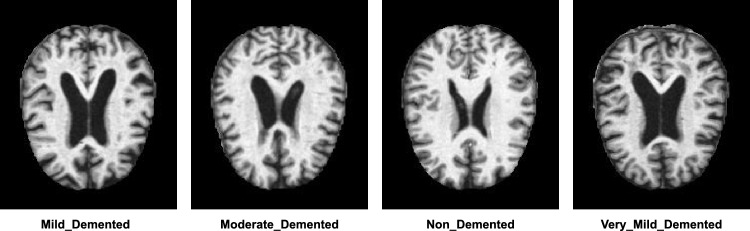
Sample MRI Images of Alzheimer’s disease based on four different cases: Mild-demented, Moderate-demented, Non-demented and Very-mild-demented.

Figure 5 illustrates the sample MRI images of four distinct cases. 80% of the total dataset samples were allocated for training, while the remaining 20% were used for testing. Table 3 shows the dataset distribution used in this study. Moreover, Table 4 compares this dataset and other widely used datasets in this field. Some sample images are shown in Fig. 5.

However, current research, such as^48^ and^49^, emphasizes subject-level data splitting to avoid overlap between training and testing datasets. These studies provide reproducible evaluation pathways for determining the robustness of Alzheimer’s disease categorization models.

Multiple safeguards were taken to avoid leaking data between the training and testing sets. To ensure similar outcomes across model executions, a constant random seed (seed=45) was utilized for repeatable data splits and model training. Data augmentation methods were only used on the training data, including resizing, denoising, zooming, shifting, rescaling, rotating, shearing, and flipping. The test set was kept separate and undisturbed until it was evaluated. The test set was entirely segregated from the training process, with no utilization of test data during training, validation, or hyperparameter tuning. This ensures that the stated performance metrics precisely represent the model’s ability to generalize to new data. In addition, K-Fold cross-validation was used on the training set to optimize hyperparameters and accurately evaluate model performance, reducing bias caused by random data splitting. These measures combined guaranteed the accuracy and trustworthiness of the data and the dependability of the performance metrics given in this study.

**Table 3:** Dataset distribution.

Cases	Training (80%)	Test (20%)	Description
Mild_Demented	717	179	Includes individuals with mild dementia who have cognitive abnormalities that are evident yet controllable
Moderate_Demented	52	12	Represents people who have substantial cognitive and functional impairment and are in the advanced stages of dementia
Non_Demented	2560	640	Individuals who show no signs of dementia
Very_Mild_Demented	1792	448	Indicates individuals who are in the early phases of cognitive impairment and have minimal symptoms

**Table 4:** Comparison of the Kaggle dataset with other widely used datasets in this domain.

Dataset	No. of Subjects	No. of Classes	Size on Desktop	Availability
OASIS^50^	416	2	1.5 GB	Need Access
ADNI^51^	822	3	5 GB	Need Access
Kaggle^47^	6400	4	32 MB	Publicly Available

### Experimental setup and runtime

The suggested architecture was executed using Keras, utilizing the TensorFlow GPU support. The experiment, including both training and testing, was conducted in the Google Colaboratory environment with Python (python3) programming language, equipped with a processor Intel Core i3-7100U CPU @ 2.4GHz, Tesla T4 graphics card, 12.67GB of RAM, and 78.19GB of disk space. Throughout the experimental procedure, the image dataset was partitioned into two separate categories: training and testing, with proportions of 80% and 20% respectively. The proposed approach is designed to classify Alzheimer’s disease into four categories: Mild-demented, Moderate-demented, Non-demented and Very-Mild-demented utilizing MRI images of the human brain.

The proposed DL architecture used Adamax optimizer, an enhancement of the Adam optimizer for deep learning, and utilizes adaptive learning rates that are adjusted dynamically throughout the training process. It employs the infinity norm for stable parameter updates and is defined by two principal parameters, beta1 and beta2, which regulate the decay rates of moment estimates. Adamax proficiently manages sparse gradients, exhibiting strong performance across many neural network structures. To maintain numerical stability throughout the parameter update phase, a small positive constant, epsilon $\epsilon$, is incorporated into the denominator to avert division by zero. This characteristic improves the reliability and efficiency of our research optimization methods, making Adamax a preferred option in our suggested deep neural network training. Our study utilized a learning rate 1e-03 and a decay rate characterized by two parameters: beta1=0.91 and beta2=0.9994. The epsilon value is also established at 1e−08. The batch size was configured at 32. The hyperparameter settings were established through experimentation, emphasizing those often utilized in similar deep learning tasks and fine-tuning according to the validation performance throughout the K-fold cross-validation procedure.

The average duration for each training epoch in this study’s extensive dataset was roughly 59 seconds, and the overall training procedure took approximately 30 minutes to reach convergence. The inference time, a crucial factor for real-time applications, was quantified to be approximately 368 milliseconds per MRI image on the NVIDIA Tesla T4 GPU. The model’s rapid inference time indicates that it is appropriate for real-time diagnostic applications, as long as sufficient processing resources exist. Again, scalability is essential when deploying in various clinical settings, including well-equipped research facilities and resource-constrained clinics. The design of the suggested model, EfficientNetV2B3, is renowned for its optimal combination of accuracy and efficiency. The model’s scalability is further improved through the fine-tuning and hyperparameter optimization conducted in this work. To assess the scalability of the model, we performed additional tests on different hardware configurations, which encompassed: The mid-range GPU option is the NVIDIA GeForce GTX 1080, while the integrated GPU option is the Intel Iris Xe Graphics. The model exhibited consistent performance across these configurations, with inference durations of 846 milliseconds on the GTX 1080 and around 51,520 milliseconds on the Intel Iris Xe. Although the model may not be optimal for real-time applications, it proves that it may nevertheless operate efficiently in less resource-intensive contexts.

### Performance metrics

The evaluation of the Alzheimer’s disease detection approach firmly depends on utilizing the confusion matrix. This indispensable tool provides a thorough overview of anticipated and observed class labels, simplifying the assessment of true-positive (TP), true-negative (TN), false positives (FP) and false negatives (FN). Presented in Table 5, this graphical depiction provides valuable information about a model’s accuracy, precision, sensitivity, f1-score and specificity which are crucial for evaluating its effectiveness across several categories. The confusion matrix facilitates model perfection and decision-making in Alzheimer’s disease detection by visually representing categorization outcomes.

**Table 5:** Confusion Matrix.

Predicted Results	Actual Positive	Actual Negative
Yes	TP	FP
No	FN	TN

Several essential performance metrics are utilized to assess the efficacy of deep learning models, each providing a unique purpose. The Eqs. 11, 12, 13, 14, 15, 16 give the mathematical formulae of these metrics including Accuracy, Precision, Sensitivity, F1-Score, Specificity and Matthewscorrelationcoefficient(MCC).

11 $$\begin{aligned} Accuracy = \frac{TP + TN}{TP + FP + FN + TN} \end{aligned}$$

12 $$\begin{aligned} Precision = \frac{TP}{TP + FP} \end{aligned}$$

13 $$\begin{aligned} Sensitivity = \frac{TP}{TP + FN} \end{aligned}$$

14 $$\begin{aligned} F1-Score = 2 \cdot \frac{(Precision \cdot Recall)}{(Precision + Recall)} \end{aligned}$$

15 $$\begin{aligned} Specificity = \frac{TN}{TN + FP} \end{aligned}$$

16 $$\begin{aligned} MCC = \frac{TP \cdot TN - FP \cdot FN}{\sqrt{(TP + FP)(TP + FN)(TN + FP)(TN + FN)}} \end{aligned}$$

In addition, this study used the loss function to evaluate the predicted model’s performance. A sparse categorical cross-entropy loss was employed to train the model. In addition to reducing the cost of the model’s parameters, the loss function was also used. Adding more epochs will lower the loss function. In terms of mathematical information, the loss function is defined by Eq. 17.

17 $$\begin{aligned} \mathcal {L}(Y, \hat{Y}) = - \left( \sum Y \cdot \log (\hat{Y}) +(1 - Y) \cdot \log \log (1 - \hat{Y}) \right) \end{aligned}$$

Here, Y = True label, $\hat{Y}$ = Predicted Labels; and L(Y,$\hat{Y}$) is loss function.

### Evaluation of the model

#### K-fold validation

K-fold coss-validation^52^ is a methodology employed to evaluate the effectiveness of a machine learning model by partitioning the dataset into multiple subsets. This study employed a five-fold cross-validation approach where 80% of the data was used for training and the rest of the 20% for validation. Specifically, in 5-fold cross-validation, the dataset is divided into five equally sized folds. The model undergoes training and assessment five times, each utilizing a distinct fold as the validation set and the remaining folds for training purposes. This iterative process ensures a comprehensive evaluation across different subsets of the data, contributing to a more robust assessment of the model’s performance.

#### Performance results

**Figure 6:**
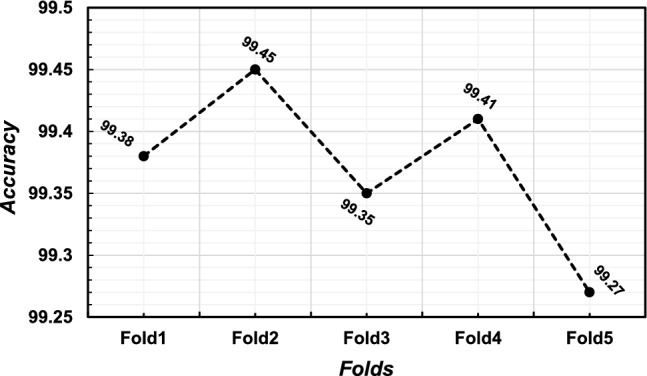
Fold-wise accuracy of the proposed model on four class categories including, Mild-demented, Moderate-demented, Non-demented and Very-mild-demented cases.

**Figure 7:**
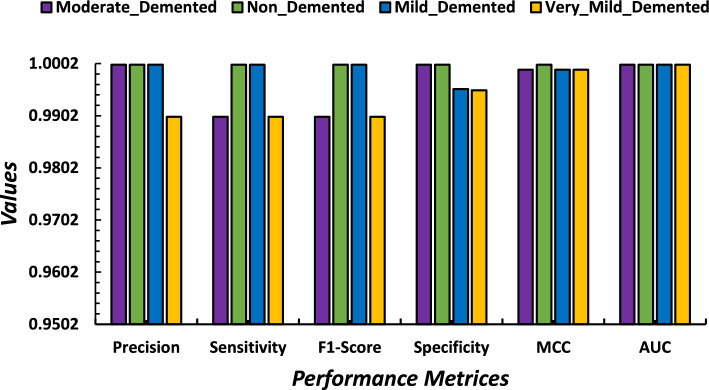
Graphical representation of performance results based on Precision, Sensitivity, F1-score, Specificity, MCC and AUC on fold-2.

**Figure 8:**
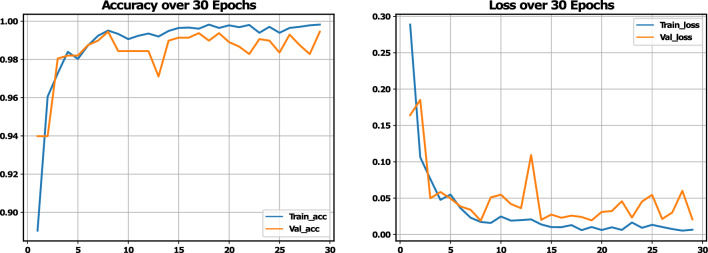
Accuracy and loss curve of the proposed model on fold-2.

**Figure 9:**
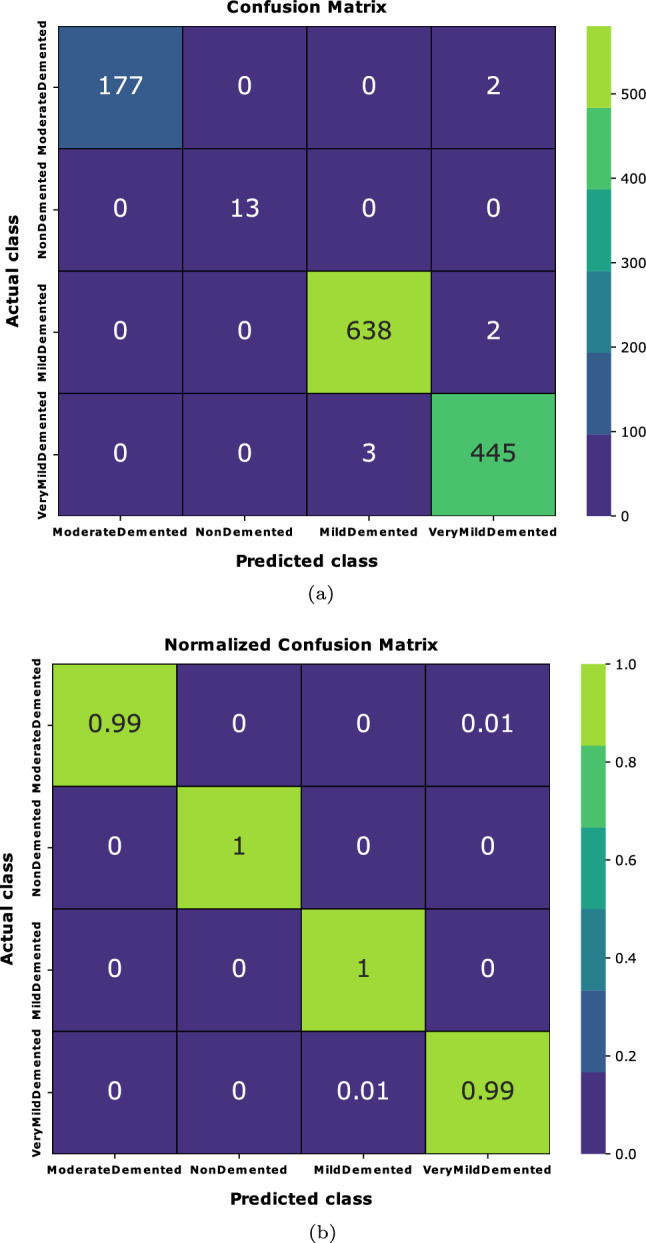
Confusion matrix of the proposed model on fold-2.

**Figure 10:**
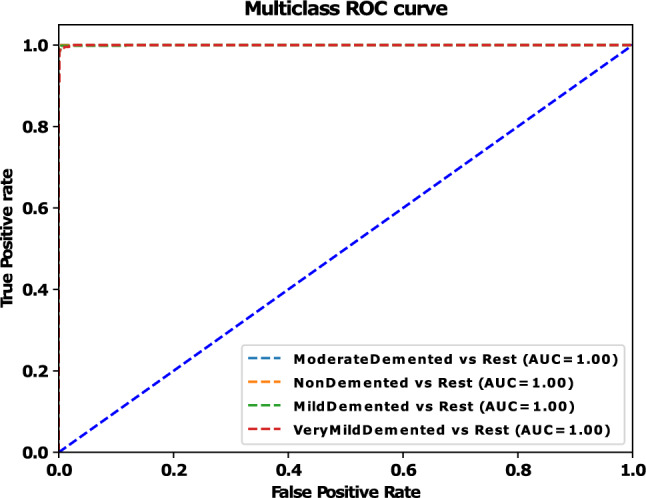
ROC curve of the proposed model using four class categories on fold-2.

A five-fold cross-validation method was employed to assess the performance results across four class categories on the Alzheimer’s disease MRI image dataset. The evaluation was conducted using the performance metrics specified in section "Performance metrics". The aggregate performance was calculated by averaging the values of each fold, as illustrated in Table 6. From Table 6, it is observed that the highest average precision, sensitivity, f1-score, and specificity are 0.9975, 0.9975, 0.995, and 0.9976, respectively, as well as the lowest average precision, sensitivity, f1-score, and specificity observed at 0.995, 0.995, 0.995, and 0.9965, respectively. Furthermore, fold-wise average accuracy results are presented in Fig. 6. The outcomes of the fold-2 classification performance using the 4-class categories including moderate demented, non demented, mild demented and very mild demented are illustrated in Fig. 7. Figure 8 depicts accuracy and loss curve results over 30 epochs on fold-2. Additionally, its confusion matrix and ROC curves are presented in Figs. 9 and 10 respectively.

The Receiver Operating Characteristic (ROC) curve is a graphical representation used to assess the performance of a classification model across different discrimination thresholds. In the context of a 4-class image classification problem for this study, the ROC curve extends its utility to multiple classes by considering each class as a separate binary classification problem against the rest. For each class, the ROC curve plots the True Positive Rate (Sensitivity) against the False Positive Rate (1 - Specificity) at various decision thresholds. The ideal scenario is represented by a curve that reaches the top-left corner, indicating perfect discrimination with a sensitivity of 1 and specificity of 1. In this specific case, where the Area Under the Curve (AUC) value for each class is reported as 1.00, it signifies a model with impeccable performance for all classes. An AUC of 1.00 implies the model achieves perfect separation between positive and negative instances, resulting in an ideal ROC curve for each class (Table 7).

**Table 6:** Performance results (precision, recall, f1-score, specificity and accuracy) of the proposed model on each fold using four class categories.

Folds	Classes	Precesion	Sensitivity	F1-Score	Specificity	Accuracy (%)
	Moderate-Demented	1.00	1.00	1.00	1.00	
Fold 1	Non-Demented	1.00	0.99	0.99	0.9935	99.38
	Mild-Demented	0.99	1.00	0.99	1.00	
	Very-Mild-Demented	1.00	0.99	1.00	0.9942	
Average		0.9975	0.995	0.995	0.9969	
	Moderate-Demented	1.00	0.99	0.99	1.00	
Fold 2	Non-Demented	1.00	1.00	1.00	1.00	99.45
	Mild-Demented	1.00	1.00	1.00	0.9953	
	Very-Mild-Demented	0.99	0.99	0.99	0.9951	
Average		0.9975	0.995	0.995	0.9976	
	Moderate-Demented	1.00	0.99	1.00	0.9932	
Fold 3	Non-Demented	0.99	1.00	1.00	1.00	99.35
	Mild-Demented	1.00	1.00	0.99	0.9948	
	Very-Mild-Demented	0.99	1.00	0.99	1.00	
Average		0.995	0.9975	0.995	0.9970	
	Moderate-Demented	1.00	0.99	0.99	1.00	
Fold 4	Non-Demented	1.00	1.00	1.00	1.00	99.41
	Mild-Demented	1.00	1.00	0.99	0.9950	
	Very-Mild-Demented	0.99	0.99	1.00	0.9953	
Average		0.9975	0.995	0.995	0.9975	
	Moderate-Demented	1.00	0.99	1.00	1.00	
Fold 5	Non-Demented	1.00	0.99	0.99	0.9925	99.27
	Mild-Demented	0.99	1.00	1.00	1.00	
	Very-Mild-Demented	0.99	1.00	0.99	0.9936	
Average		0.995	0.995	0.995	0.9965

**Table 7:** Performance results based on Precision, Recall, F1-Score, Specificity, MCC, AUC and Accuracy of the proposed model on fold-2 using four class categories.

Classes	Precesion	Sensitivity	F1-Score	Specificity	MCC	AUC	Acc. (%)
Moderate-Demented	1.00	0.99	0.99	1.00	0.999	1.00	
Non-Demented	1.00	1.00	1.00	1.00	1.00	1.00	99.45
Mild-Demented	1.00	1.00	1.00	0.9953	0.999	1.00	
Very-Mild-Demented	0.99	0.99	0.99	0.9951	0.999	1.00	
Average	0.9975	0.995	0.995	0.9976	0.99925	1.00	

#### Discriminative localization by Proposed Model Using Grad-Cam++

**Figure 11:**
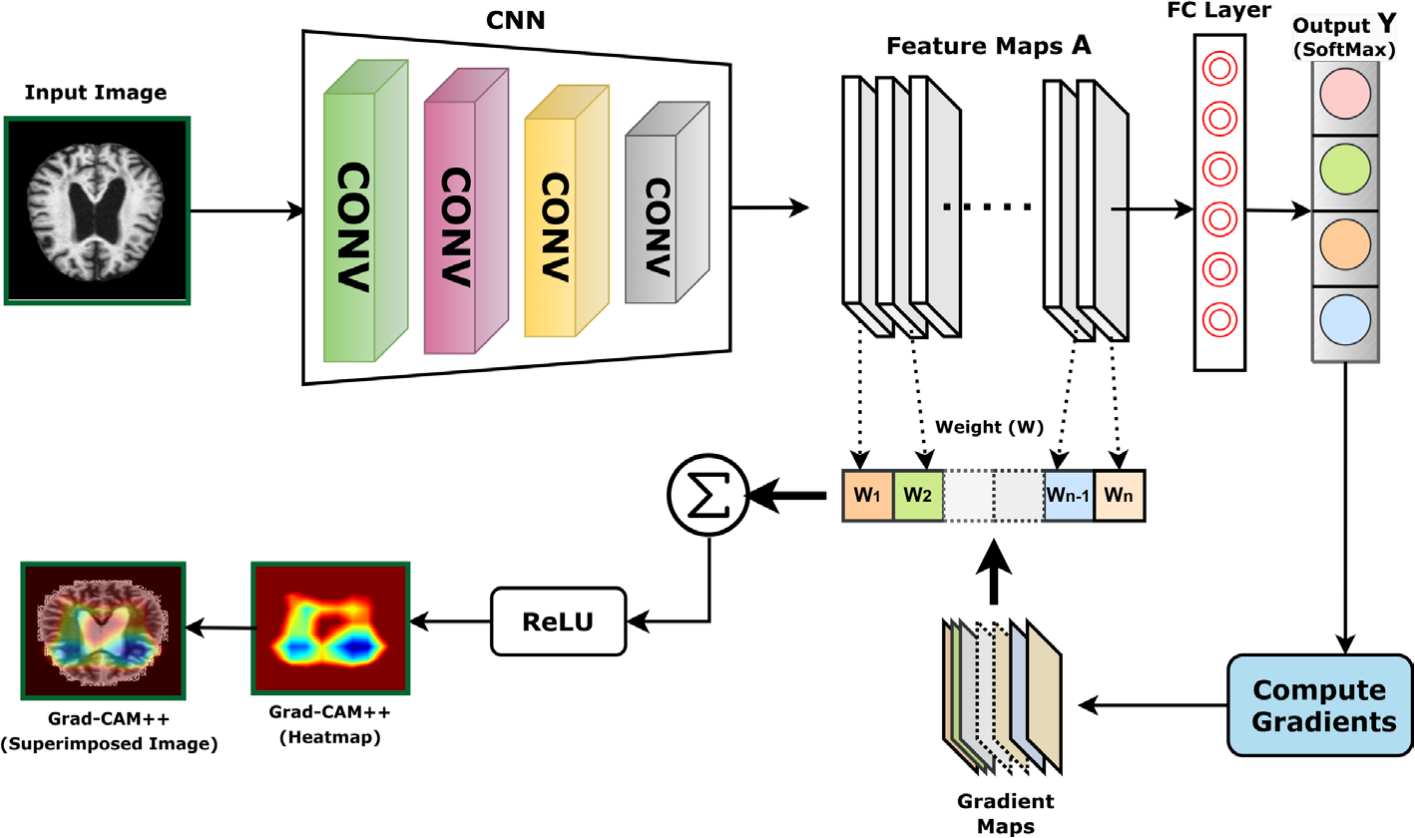
Procedure of gradient weighted class activation mapping++ (Grad-CAM++) as follows: The final feature map’s gradient of the target output class is utilized to localize significant portions coarsely.

Grad-CAM++ (Gradient-weighted Class Activation Mapping++) is an extension of the Grad-CAM technique used in computer vision for visualizing and understanding convolutional neural networks (CNNs)^53^. Our proposed model incorporates Gradient-based class activation mapping++ (Grad-CAM++) to provide class activation mapping for identifying the particular region of the MRI images that mostly influenced the decision, as illustrated in Fig. 11. After processing through the final layer, a preliminary localization map is created to identify the key areas within the image for prediction. It helps interpret the decision-making process of deep learning models by highlighting the regions of input images that contribute the most to the model’s predictions. Unlike the original Grad-CAM, Grad-CAM++ considers both positive and negative gradients to localize essential features in the input image better. This enhanced technique provides more accurate and precise visual explanations, aiding researchers and practitioners in gaining insights into the inner workings of CNNs and improving model transparency and interpretability.

**Figure 12:**
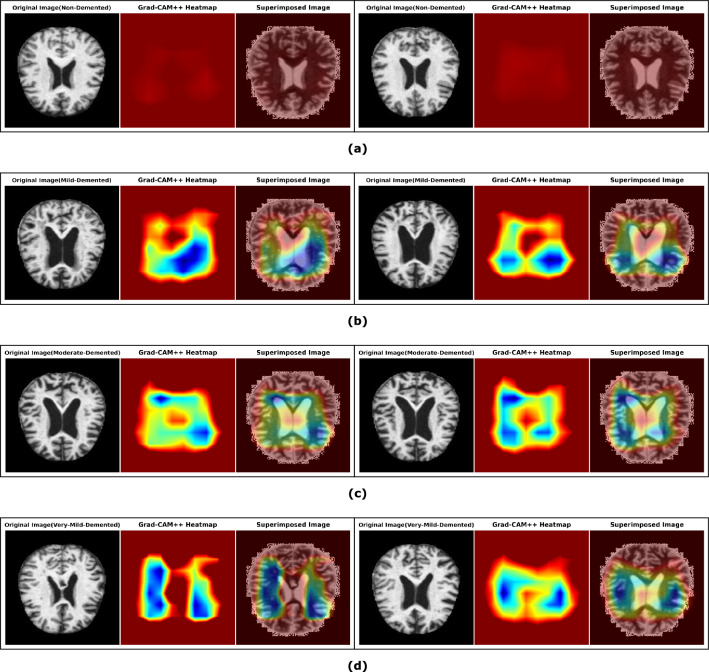
Key sections of the MRI images (before and after scenario) that drive the decision are identified by applying the class activation heatmap (Grad-CAM++) generated by the proposed model: (**a**) non demented (**b**) mild demented (**c**) moderate demented and (**d**) very mild demented.

The primary enhancement of Grad-CAM++ is in its methodology for computing the significance weights assigned to each feature map. Below is an elaborate elucidation of the equations employed in Grad-CAM++:

i. Compute the Grad-CAM++ weights: Given a feature map $A^k$ (where $k$ denotes the index of the feature map) and the gradient of the score for a sample class $c$ (represented as $y^c$) with respect to the feature map $A^k$, the weight $\alpha _{ij}^k$ is calculated as follows in Eq. 18.
  18 $$\begin{aligned} \alpha _{ij}^k = \frac{\frac{\partial ^2 y^c}{\partial (A_{ij}^k)^2}}{2 \frac{\partial ^2 y^c}{\partial (A_{ij}^k)^2} + \sum _a \sum _b A_{ab}^k \cdot \frac{\partial ^3 y^c}{\partial (A_{ij}^k)^3}} \end{aligned}$$
  Where, The terms $\frac{\partial ^2 y^c}{\partial (A_{ij}^k)^2}$ and $\frac{\partial ^3 y^c}{\partial (A_{ij}^k)^3}$ represents the second-order and third-order partial derivative of the class score $y^c$ with respect to the activation $A_{ij}^k$ at the spatial location $(i, j)$ in the feature map $k$ respectively.
ii. Compute the Grad-CAM++ maps: The Grad-CAM++ map $L_{\text {Grad-CAM++}}^c$ is subsequently derived by integrating the feature maps with the calculated weights is represented in Eq. 19.
  19 $$\begin{aligned} L_{\text {Grad-CAM++}}^c = \sum _{k} \alpha ^k \cdot \text {ReLU} \left( \sum _{i} \sum _{j} \alpha _{ij}^k \cdot \frac{\partial y^c}{\partial A_{ij}^k} \right) \end{aligned}$$
  where, The weights $\alpha _k$ are obtained by summing over $\alpha _{ij}^k$ for all $i$ and $j$ for the feature map $k$, represented as: $\alpha _k = \sum _{i} \sum _{j} \alpha _{ij}^k$. Additionally, the ReLU($\cdot$) function, which stands for Rectified Linear Unit, guarantees that only positive values contribute to the map. These weights generate a heatmap identifying the crucial areas in the input image that the model considers significant for its decision-making for a particular class. Some of these localizations are explored further by overlaying the heatmap on the input MRI images to assess the network’s learning from a clinical perspective. Figure 12 shows some MRI images with imposed localization. The intense blue color indicates the most critical region (processed attributes) from which the network has made the classification decision. Here are a few key findings:

- Non-demented MRI images of the brain offer valuable insights into the brain’s structural integrity and normal functioning. According to the study^54^ they found age-related changes in various brain regions, including white matter maturation and cortical thickness, highlighting the importance of non-demented MRI data in understanding typical brain development. From Fig. 12a we found no substantial zone is localized.
- Upon analyzing the heatmaps for cases of mild dementia from Fig. 12b, it is evident that our suggested model has identified and highlighted the specific regions associated with the hippocampus and entorhinal cortex^55,56^.
- Moderate dementia, a stage of neurodegenerative diseases like Alzheimer’s, is associated with significant structural changes in the brain that can be visualized through MRI (Magnetic Resonance Imaging). Upon examining the heatmap depicted in Fig. 12c, it is apparent particularly in regions implicated in memory and cognition, such as the hippocampus, entorhinal cortex, and frontal lobes^57^.
- Very mild dementia, presents a crucial aspect in understanding the early stages of neurodegenerative diseases such as Alzheimer’s. By analyzing the heatmap from Fig. 12d, it is observed that there may be subtle alterations in the volume and integrity of specific brain regions associated with memory and cognitive function, such as the hippocampus and cortical areas^58^.

**Figure 13:**
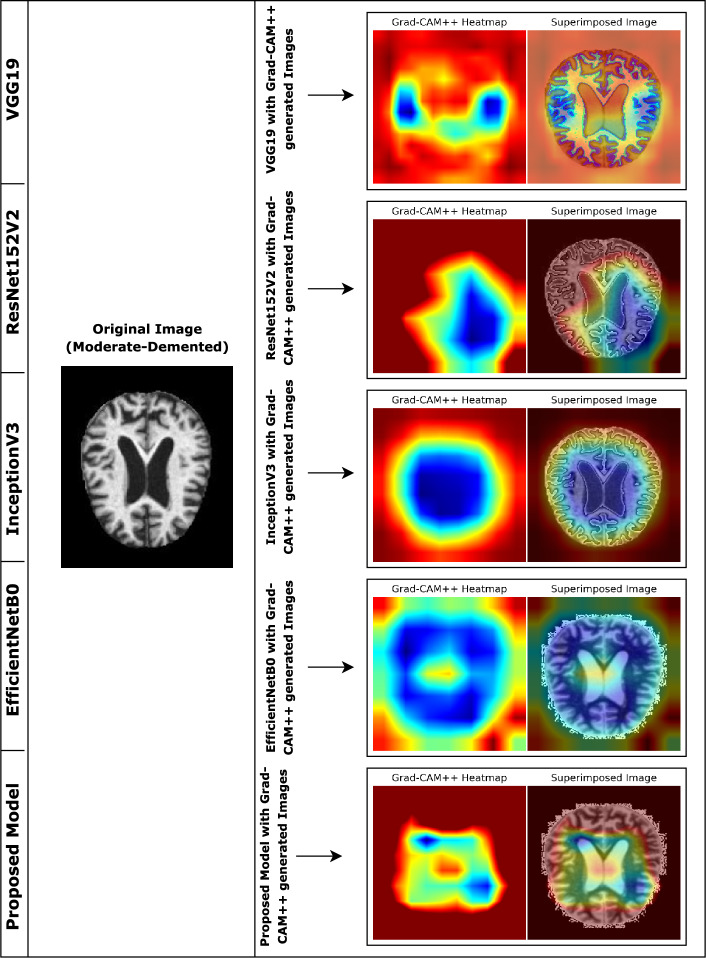
Grad-CAM++ generated images of different pretrained models and the proposed model on a sample image of Moderate Dementia case.

As the proposed model is incorporated with Grad-CAM++ and we also compare the model with other previous pretrained models based on various aspects, as shown in Table 8. Furthermore, Fig. 13 presents the outcome of different models with grad-cam++ and the proposed model with grad-cam++ by considering a sample image of a moderate dementia case.

**Table 8:** Comparison between the proposed model and the other pretrained models based on various aspects.

Aspect	VGG19	ResNet152V2	InceptionV3	EfficientNetB0	Proposed Model
Parameter	More parameters	More parameters	More parameters	Less parameters	Fewer parameters
	($\tilde{1}$43.7M)	($\tilde{6}$0.4M)	($\tilde{2}$3.9M)	($\tilde{5}$.3M)	($\tilde{1}$4.5M)
Model	Larger, generating	Larger model size,	Larger, leading to	Smaller model than	Smaller, facilitating
Size	slower inference	slower inference	slower inference	EfficientNetV2B3	faster inference
Training	Longer due to	Moderate training	Longer due to	Shorter due to	Typically shorter for
Time	increased number	time	more parameters	fewer parameters	fewer parameters
	of parameters				
Feature	Effective but less	Effective at	Effective but not	Efficient, but less	More effective at
Extraction	efficient than	extracting	as efficient	so compared	obtaining relevant
	EfficientNetV2B3	relevant features	as EfficientNetV2B3	to EfficientNetV2B3	features from MRIscans
Heatmap	Generate less precise	Detailed heatmaps	Detailed heatmaps	Generates precise	Generates more
Precision	heatmaps than	but less refined	but less refined	heatmaps but less	refined and
	EfficientNetV2B3			detailed than V2B3	precise heatmaps
Interpret-	Good; but, heatmaps	High interpretability	High, but heatmaps	High, but less refined	Higher due to refined
ability	can have less detail	with	can be less detailed	than V2B3	heatmaps, making it easier
		detailed heatmaps			to interpret brain regions
Scalability	Less scalable compared	Good scalability	Good scalability	Good scalability	Better scalability with
	to EfficientNetV2B3				compound scaling

According to Fig. 13, the heatmap and superimposed images generated by the proposed model are more refined and precise than others.

## Discussion

This section critically evaluates our findings, compares them with prior research, and examines the implications and constraints of our suggested model.

### Critical analysis of findings

This section assesses the performance of our proposed model, highlighting its significance accuracy (99.45%), precision (99.75%), specificity (99.76%), recall (99.5%), MCC (99.92%) and AUC (100%) in classifying Alzheimer’s disease into four categories: mild, moderate, very mild, and non-demented and integrates it with the distinct hybrid filtering method and the enhanced EfficientNetV2B3 architecture. Additionally, it examines the Grad-CAM++ outcomes and their clinical significance. Thus, the study shows how the hybrid filtering strategy and modified EfficientNetV2B3 architecture strengthened the model and improved classification performance.

### Comparison with previous studies

The findings of this study were compared to those of other recent state-of-the-art works in this field and other pre-trained models. Initially, the suggested model was contrasted with several prior trained models. In this case, four different classes were considered for comparisons-with and without prepossessing on image data. Table 9 shows the comparisons between the suggested and the other pre-trained models. Therefore, it is observed from Table 9 that models trained without utilizing preprocessed data exhibit lower accuracy, precision, sensitivity, f1-score, and specificity values in comparison to models trained with preprocessed data. In contrast, the suggested model demonstrated a remarkable accuracy of 99.45%. In addition, the proposed model produced substantial scores for precision, sensitivity, f1-score and specificity as shown in Table 9. Therefore, the suggested architecture may significantly enhance its effectiveness and robustness by implementing efficient preprocessing techniques on image data and utilizing the pre-trained “EfficientNetV2B3” model. Again, the proposed model exhibits a remarkable accuracy of 97.67% without preprocessing, in contrast to a pretrained model like VGG16’s 92.41% with preprocessing (Table 9); nevertheless, the unique properties of the Kaggle dataset may have impacted these outcomes. The dataset’s intrinsic quality, structure, and distribution may partially exaggerate the model’s performance. To ascertain the robustness and generalizability of the suggested methodology, subsequent research should entail testing on varied datasets, including ADNI and OASIS. An ablation research examining the influence of preprocessing approaches on model performance will elucidate their effects.

To statistically validate the performance of the proposed model, confidence intervals (CI) provide a reliable metric to assess its accuracy compared to baseline methods. The computed 95% confidence interval (CI = [99.05%, 99.86%]) suggests that the model exhibits consistently high accuracy, demonstrating its robustness and reliability. This narrow interval signifies a low variability in classification results, reinforcing the model’s consistency.

**Table 9:** Evaluation of the proposed model in relation to other pre-trained models.

DL_Models	Preprocessing	Precision	Sensitivity	F1-score	Specificity	Acc(%)
	on Image Data					
Vgg16	No	0.894	0.902	0.893	0.895	89.25
	Yes	0.923	0.915	0.917	0.923	92.41
ResNet101V2	No	0.925	0.930	0.927	0.932	93.35
	Yes	0.953	0.947	0.954	0.948	95.17
InceptionResNetV2	No	0.952	0.945	0.945	0.953	95.33
	Yes	0.974	0.967	0.967	0.975	97.54
MobileNetV2	No	0.904	0.896	0.895	0.903	90.25
	Yes	0.934	0.928	0.926	0.935	93.53
DenseNet201	No	0.935	0.928	0.933	0.935	93.64
	Yes	0.957	0.951	0.955	0.957	95.52
EfficientNetB0	No	0.933	0.926	0.931	0.927	93.43
	Yes	0.955	0.948	0.953	0.948	95.31
EfficientNetB3	No	0.956	0.952	0.947	0.954	95.67
	Yes	0.977	0.975	0.968	0.976	97.82
EfficientNetV2B0	No	0.942	0.941	0.937	0.943	94.25
	Yes	0.964	0.962	0.958	0.965	96.47
EfficientNetV2B1	No	0.952	0.946	0.953	0.954	95.31
	Yes	0.974	0.967	0.975	0.977	97.53
Proposed Model	No	0.975	0.967	0.977	0.979	97.67
(Modified_EfficientNetV2B3)	Yes	0.997	0.995	0.995	0.997	99.45

The evaluation outcomes of the suggested investigation were also contrasted with prior examples of comparable recent studies by considering their architecture, number of classes and subjects. As shown in Table 10, the proposed research achieved 99.45% accuracy for four class categories consisting of mildly demented, moderately demented, non-demented, and very mildly demented cases. Agarwal et al.^59^ introduced a novel approach, combining end-to-end and transfer learning, utilizing the EfficientNet-b0 convolutional neural network (CNN). By analyzing 245 T1W MRI scans of cognitively normal (CN) subjects, 229 AD subjects, and 229 subjects with stable mild cognitive impairment (sMCI), the study achieved promising results in classification tasks.

Shankar et al.^60^ employed a thorough methodology for detecting Alzheimer’s disease, which included preprocessing images and extracting features using texture scale invariants, transforms, and histograms. They augmented the performance of classifiers such as CNN, KNN, and decision trees by applying Group Grey Wolf Optimization (GGWO) techniques. This approach yielded a remarkable accuracy of 96.23% in Alzheimer’s detection, surpassing the effectiveness of alternative methods. The above study did not utilize additional patient data and only considered three class categories. Nevertheless, our proposed model included a substantial quantity of patient data consisting of MRI images, categorized into four classes for the experiment. As demonstrated in Table 10, we achieved accuracy beyond the results reported in these investigations. Although Yang et al.^61^ and Pradhan et al.^62^ examined four different case types for the detection of Alzheimer’s disease, their collected dataset and accuracy were considerably lower than those of our proposed study, as indicated in Table 10. Additionally, they only considered traditional deep learning pretrained models, whereas we utilized a substantial number of MRI images and developed an advanced pretrained model. Zaabi et al.^63^ proposed a two-step approach: first, extracting regions of interest (e.g., the hippocampus) from brain images, followed by classification using deep learning techniques like Convolutional Neural Networks (CNN) and Transfer Learning. Evaluation on the Oasis dataset demonstrates Transfer Learning’s superior performance, achieving a classification rate of 92%.

**Table 10:** Comparison of the proposed model with existing models for the multi-class categorization task.

Study	Architecture	No. of class	F1-score	Specificity	AUC	Acc (%)
		and Subjects				
^63^	CNN +	2, AD and Healthy	x	x	x	92.86
	Transfer Learning	and Control (HC)				
^64^	Siamese Network	2, mild cognitive	0.907	0.929	x	92.72
		impairment (MCI) and AD				
^59^	EfficientNet-b0	3, cognitively normal (CN),	0.8643	x	0.910	87.38
		AD, stable mild cognitive				
		impairment (sMCI)				
^60^	GGWO techniques	3, mild cognitive impairment	x	0.9623	x	96.23
		(MCI), healthy control (HC)				
		and AD				
^61^	VGG19	4, Non Demented (ND),	0.987	x	0.998	98.6
		Very Mild Demented (MD),				
		Mild Demented (MD) and				
		Moderated -Demented (MDTD)				
^62^	VGG19 +	4, Mildly, Moderately,	x	x	x	94.00
	DenseNet169	Very Mildly and Non-Demented				
Proposed	Modified	4, Mild Demented, Moderate	0.995	0.9976	1.00	99.45
Model	EfficientNetV2B3	Demented, Non Demented				
		and Very Mild Demented				

Razavi et al.^64^ proposed a novel two-stage method integrating unsupervised feature learning with SoftMax regression for intelligent diagnosis. Evaluation on Alzheimer’s Brain image datasets offered automation and adaptability for efficient big data processing, as evidenced by experiments on ADNI data. The aforementioned study did not perform any sort of data preprocessing or augmentation on their raw data. Also, the dataset they chose was significantly less than the one we used in this study. The suggested model in this research used both preprocessing and augmentation methods on the raw images, which improved the outcomes as shown in Table 10, because this improves and reinforces the deep learning model.

**Table 11:** Comparison of the proposed model with recently published state-of-the-art works on several datasets.

Study	Architecture	Database &	Performance
		No. of Classes	
Hazarika et al.	Hybrid pretrained	ADNI & Three	Accuracy = 88%
^29^	models		Precision = 92%
			Recall = 90%
			F1-score = 91%
Balaji et al.	CNN + LSTM	Kaggle & Two	Accuracy = 98.50%
^38^			Precision = 94.80%
			Recall = 98%
Hu et al.	Pretrain model +	ADNI & Two	Accuracy = 77.20%
^39^	Transformer		Sensitivity = 79.97%
			Specificity = 71.59%
Sethuraman et al.	Hybrid pretrained	ADNI & Two	Accuracy = 96.61%
^40^	models		Sensitivity = 94.34%
			Specificity = 94.96%
Marwa et al.	CNN	OASIS & Two	Accuracy = 99.68%
^30^			
El-Latif et al.	Lightweight CNN	Kaggle & Four	Accuracy = 95.93%
^14^			Precision = 95.93%
			Recall = 95.88%
			F1-score = 95.90%
Suchitra et al.	EfficientNetB7	ADNI & Multi	Accuracy = 98.2%
^32^			Sensitivity = 98.08%
			Specificity = 98%
			F1-score = 98.95%
Mahmud et al.	Ensemble DL models	Kaggle & Four	Accuracy = 96%
^37^			Precision = 89%
			Recall = 93%
			F1-score = 91%
E. Kina	Attention DL model	Kaggle & Four	Accuracy = 95.19%
^34^			
Proposed	Modified	Kaggle & Four	Accuracy = 99.45%
Model	EfficientNetV2B3		Precision = 99.75%
			Recall = 99.5%
			F1-score = 99.5%
			Specificity = 99.76%

Furthermore, we also compared our model with recently published works on several datasets as shown in Table 11. The suggested model, utilizing the modified EfficientNetV2B3 architecture, surpasses existing models regarding accuracy, precision, specificity, and recall. These findings indicate that the proposed modifications and the selection of EfficientNetV2B3 are highly successful in accomplishing the classification tasks on the Kaggle/DUBEY (2020) dataset. The comparative analysis highlights the proposed model’s strength and effectiveness compared to recent cutting-edge models as shown in Table 11. Although Suchitra et al.^32^ attained 98.2% accuracy utilizing EfficientNetB7, our suggested model employing the improved EfficientNetV2B3 architecture obtained 99.45% accuracy. The enhancement is due to EfficientNetV2B3’s optimized training methodology, superior regularization techniques, and advanced feature extraction capabilities, enabling it to identify more pertinent patterns in MRI images. Moreover, EfficientNetV2B3 possesses fewer parameters than EfficientNetB7, resulting in diminished computational expenses.

The proposed model attains an accuracy of 99.45%; nevertheless, employing EfficientNetV2B3 with additional layers escalates the model’s complexity and poses a risk of overfitting. To limit this risk, we utilized various strategies, including data augmentation (rotation, shifting, zooming, and flipping), dropout layers (with rates of 0.2 and 0.3), and K-fold cross-validation. Examining the training and validation curves (Fig. 8) indicates that the curves are closely aligned, implying that the model is generalizing effectively and not exhibiting overfitting.

Moreover, for discriminative visualization of our findings on this MRI image dataset we used an advanced explainable AI technique named Grad-CAM++.Furthermore, we compared our model and both Grad-CAM++ and the baseline model (Grad-CAM).

Significantly, Grad-CAM++ improves upon Grad-CAM by incorporating additional information from deeper neural network layers, resulting in more precise localization of important regions within an image.

**Figure 14:**
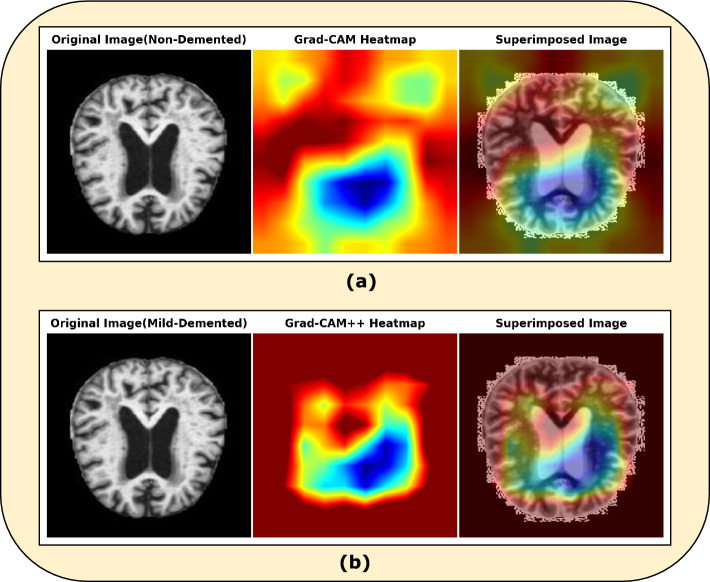
Grad-CAM and Grad-CAM++ generated images of the proposed model on a sample image of Mild Dementia case.

- Differences: The main difference between the two images as presented in Fig. 14 lies in the precision and localization of the heatmaps generated by Grad-CAM and Grad-CAM++ with our proposed model.
  i. Grad-CAM generated Image Fig. 14a: The heatmap highlights a broader area, indicating that a larger region of the original image influences the model’s prediction. This suggests the model might consider a more general pattern or feature within the image.
  ii. Grad-CAM++ generated Image Fig. 14b: The heatmap is more focused and precise, highlighting a smaller, more specific region of the image. This indicates the model relies on a more localized feature or detail within the image to predict.

The proposed model utilizing Grad-CAM++ for localization demonstrates several superiorities over the models discussed in the following selected articles. Firstly, compared to^36^, who used an NCA-based hybrid CNN model with Grad-CAM, our model provides more precise localization of Alzheimer’s disease-related regions in brain MRI images. This precision is evident from the more focused and detailed heatmaps produced by Grad-CAM++, as shown in Fig. 14b- superimposed image. Secondly, in contrast to the approach by^31^, which integrates XAI with Grad-CAM, our model’s use of Grad-CAM++ offers enhanced interpretability and finer granularity in highlighting significant areas, aiding clinicians in making more accurate diagnoses. Additionally, Raju et al.^65^ employed a deep learning-based multilevel classification system; however, our model’s improved localization capabilities can potentially lead to higher classification accuracy by focusing on the most relevant features.

In essence, Grad-CAM++ provides a more fine-grained and accurate localization of the important regions in the image, making it easier to understand which parts of the image are driving the model’s decision. This makes our model a more effective tool for early disease progression detection and monitoring.

### Significance of the model

#### Evaluation of research queries

- **RQ1**: How well does the proposed hybrid filtering method enhance MRI image preprocessing to strengthen model robustness and improve accuracy?
  **Evaluation:** The proposed hybrid filtering method enhances MRI image preprocessing by combining adaptive non-local means denoising and sharpening filters to effectively reduce noise while preserving fine details and improving edge definition. This preprocessing approach strengthens model robustness by optimizing image quality, leading to improved feature extraction and achieving a high classification accuracy of 99.45% as shown in Table9.
- **RQ2**: How does the proposed model perform relative to other state-of-the-art methods in terms of accuracy, precision, recall, and specificity for multi-class Alzheimer’s disease diagnosis?
  **Evaluation:** The proposed model, leveraging a modified EfficientNetV2B3 architecture, achieves a high accuracy of 99.45% for four-class Alzheimer’s disease diagnosis, outperforming existing models like VGG19 and ResNet152V2. It also demonstrates superior precision (99.75%), recall (99.5%), and specificity (99.76%) compared to other state-of-the-art methods shown in Table 9, 10 and 11 indicating its effectiveness in accurately classifying different stages of Alzheimer’s disease from MRI images. This performance is attributed to the model’s efficient feature extraction, fine-tuning techniques, and preprocessed image data.
- **RQ3**: How do advanced explainable AI techniques, such as Grad-CAM++, enhance clinicians’ ability to interpret and trust AI-based diagnostic approaches?
  **Evaluation:** Advanced explainable AI techniques, such as Grad-CAM++, enhance clinicians’ ability to interpret and trust AI-based diagnostic approaches by providing detailed visual explanations of the specific regions in medical images that influence the model’s predictions. As demonstrated in the study, this improved localization and precision allow clinicians to validate AI decisions against clinical knowledge, increasing transparency and confidence in the diagnostic process.

#### Clinical implementation considerations

The proposed study significantly impacts healthcare by improving early Alzheimer’s detection through a deep transfer learning model, allowing for timely interventions and better patient outcomes. Its high accuracy of 99.45% ensures reliable diagnostics, offering a trustworthy tool for healthcare professionals. Utilizing publicly available MRI datasets and transfer learning enhances resource efficiency, making it accessible for under-resourced settings. The inclusion of Grad-CAM++ facilitates explainable AI, helping clinicians understand and trust the diagnostic process. The methodology’s adaptability to other neurodegenerative diseases also broadens its potential impact across medical diagnostics.

### Limitations and future directions

Although the proposed approach has shown remarkable success in classifying Alzheimer’s disease using MRI images, it is crucial to recognize the constraints of the dataset employed in this investigation. The dataset utilized in this study demonstrates a class imbalance, characterized by a limited number of samples in the Moderate Demented group (64 samples). This imbalance may bias the model towards the majority classes, impairing its capacity to categorize Moderate Demented cases reliably. To address this issue, we will investigate advanced data augmentation approaches. Future research will investigate GAN-based approaches to mitigate class imbalance further and enhance classification accuracy for the Moderate Demented category. Additionally, the dataset may lack complete representativeness of the worldwide population due to its acquisition from publicly accessible archives and the incomplete/imbalanced disclosure of demographic information on the participants. This could potentially add bias and impact the generalizability of the model. Furthermore, the variability in image quality of MRI scans from different sources can influence the model’s performance. To overcome these constraints, future research may involve collecting a broader and more diverse dataset, encompassing comprehensive demographic data and maintaining constant image quality and balancing. In addition, conducting a more extensive assessment of the model’s effectiveness across various demographics and environments would be beneficial.

The model’s efficient architecture and rapid inference times on high-performance GPUs highlight its potential for real-time clinical applications. Future efforts will aim to integrate the model with existing clinical workflows and imaging software, ensuring user-friendly interfaces and broad adoption in medical diagnostics.

Overall, the positive and hopeful outcomes of our suggested model in detecting Alzheimer’s disease from MRI images indicate that deep transfer learning could significantly impact fighting the ongoing threat in the coming years.

## Conclusions

In this study, we proposed a deep transfer learning model that presents a promising solution for the timely identification of Alzheimer’s disease (AD) through the analysis of brain MRI images. The model, leveraging EfficientNetV2B3 architecture and fine-tuning with additional layers, demonstrates a high accuracy rate of 99.45% in classifying cases of Mild demented, normal, Moderate demented, and Very Mild demented. By incorporating Grad-CAM++ for discriminative localization, the model accurately identifies AD pathology and provides interpretable insights into radiological anomalies associated with the disease. These findings underscore the potential of sophisticated transfer learning techniques in augmenting AD diagnosis, particularly in resource-constrained settings. Ultimately, the proposed model holds promise for assisting healthcare providers in prompt identification and intervention, thereby improving patient outcomes in this pressing global health concern.

**Limitation:** Despite this study’s promising results, certain limitations remain. First, the research focuses on deep transfer learning; other machine learning techniques were not explored. Second, attention mechanisms, such as self-attention or transformer-based models, were not incorporated. Third, the model has not been integrated within a clinical device, which restricts its immediate applicability in practical healthcare settings.

**Future work:** Building upon the limitations identified in this study, future work will focus on addressing the following key aspects. Firstly, other machine learning techniques, including conventional and hybrid approaches. Secondly, attention mechanisms, such as self-attention and transformer-based architectures, will enhance the model’s ability to capture intricate relationships and dependencies within MRI image data. Finally, efforts will be made to integrate the proposed model within clinical devices and real-world workflows. This step will involve rigorous testing in clinical environments to assess its usability, reliability, and overall impact on patient outcomes.

## Data Availability

The Dataset is collected from Kaggle: Alzheimer’s Dataset: https://www.kaggle.com/datasets/yasserhessein/dataset-alzheimer

## Appendix

See Table 12.

**Table 12:** Definition of mathematical symbols with context/usage in the proposed study.

**Symbol**	**Definition**	**Context/Usage**
*I*	Input image affected by noise	Image preprocessing (denoising using NLM filtering)
*u*	Pixel values in the noisy image	Used in Equation (1) as input
*v*	Pixel values after denoising	Output of NLM filtering
*x*	Position of a pixel in the image	Filtering equations
*y*	Position of a neighboring pixel	Weighting in NLM filtering
$\Omega$	Neighborhood around pixel *x*	Defines averaging area
*w*(*x*, *y*)	Weight between pixels *x* and *y*	Determines contribution in denoising
*C*(*x*)	Normalization factor for weights	Ensures weights sum to 1
*P*(*x*)	Patch centered at *x*	For similarity computation in NLM filtering
*h*	Filtering strength parameter	Controls decay in Gaussian kernel (Equation 2)
*Z*(*x*)	Normalization constant in weight calculation	For Gaussian kernel normalization
*f*(*x*, *y*)	Pixel intensity at (*x*, *y*)	Used in sharpening (Laplacian) filters
$\nabla ^2 f$	Laplacian of image *f*	Second-order derivative for sharpening
*g*(*x*, *y*)	Output pixel after sharpening	Final result of Laplacian-based sharpening
$W_1, \ldots , W_9$	Pixel weights in sharpening filter mask	Part of filter kernel
TP, TN, FP, FN	True Positives, True Negatives, False Positives, False Negatives	Performance metrics
Accuracy	Overall correctness of prediction	Performance evaluation
Precision	Proportion of true positive predictions	Performance evaluation
Sensitivity / Recall	Ability to detect positive instances	Performance evaluation
F1 Score	Harmonic mean of precision and recall	Performance evaluation
Specificity	Ability to detect negative instances	Performance evaluation
Matthews Correlation Coefficient (MCC)	Measure of the quality of binary and multiple classifications	Performance evaluation
$L(Y, \hat{Y})$	Loss function	Sparse categorical cross-entropy
*Y*	True label	Used in loss calculation
$\hat{Y}$	Predicted label	Used in loss calculation
$\alpha ^k_{ij}$	Grad-CAM++ weight at location (*i*, *j*) in feature map *k*	Used in explanation of model’s prediction
$y^c$	Score for class *c*	Class output score used in Grad-CAM++
$A^k_{ij}$	Activation at pixel (*i*, *j*) in feature map *k*	Intermediate CNN output
$L^c_{\text {Grad-CAM++}}$	Grad-CAM++ localization map for class *c*	Visualization of important regions in MRI
ReLU	Rectified Linear Unit activation	Used in CNNs and Grad-CAM++
